# Early Detection of *Fusarium oxysporum* Infection in Processing Tomatoes (*Solanum lycopersicum*) and Pathogen–Soil Interactions Using a Low-Cost Portable Electronic Nose and Machine Learning Modeling

**DOI:** 10.3390/s22228645

**Published:** 2022-11-09

**Authors:** Hanyue Feng, Claudia Gonzalez Viejo, Niloofar Vaghefi, Paul W. J. Taylor, Eden Tongson, Sigfredo Fuentes

**Affiliations:** 1 Digital Agriculture Food and Wine Group, School of Agriculture and Food, Faculty of Veterinary and Agricultural Sciences, The University of Melbourne, Parkville, VIC 3010, Australia; 2 School of Agriculture and Food, Faculty of Veterinary and Agricultural Sciences, The University of Melbourne, Parkville, VIC 3010, Australia

**Keywords:** collar and root rot, soilborne pathogens, yield decline, volatile compounds, plant disease modeling, artificial neural networks

## Abstract

The early detection of pathogen infections in plants has become an important aspect of integrated disease management. Although previous research demonstrated the idea of applying digital technologies to monitor and predict plant health status, there is no effective system for detecting pathogen infection before symptomatology appears. This paper presents the use of a low-cost and portable electronic nose coupled with machine learning (ML) models for early disease detection. Several artificial neural network models were developed to predict plant physiological data and classify processing tomato plants and soil samples according to different levels of pathogen inoculum by using e-nose outputs as inputs, plant physiological data, and the level of infection as targets. Results showed that the pattern recognition models based on different infection levels had an overall accuracy of 94.4–96.8% for tomato plants and between 94.81% and 96.22% for soil samples. For the prediction of plant physiological parameters (photosynthesis, stomatal conductance, and transpiration) using regression models or tomato plants, the overall correlation coefficient was 0.97–0.99, with very significant slope values in the range 0.97–1. The performance of all models shows no signs of under or overfitting. It is hence proven accurate and valid to use the electronic nose coupled with ML modeling for effective early disease detection of processing tomatoes and could also be further implemented to monitor other abiotic and biotic stressors.

## 1. Introduction

The Australian Processing Tomato Industry (APTI) has been affected by yield decline over recent years, which is partly attributed to the increase in disease incidence and severity caused by a novel *Fusarium oxysporum* that causes collar and root rot. *F. oxysporum* is a slow-growing species compared to many *Pythium* species that co-existed with it in processing tomatoes, and it often causes severe damage in the middle to late stages of the host plants [1,2,3]. Affected plants usually had poor root development and could not reach full growth and production potential. In more severe cases, approximately 10–15% of the processing tomato plants were infected [1,2]. The APTI depends on field surveys to assess the disease incidence based on symptomology. For general isolation and identification of putative soilborne pathogens that have been widely applied, diseased plants are sent to professional laboratories to confirm pathogen identification through culturing and DNA sequences [1,2,3]. Although various pathogens of processing tomatoes have been successfully isolated and identified through this general approach, disease detection occurs after plants have been infected and are exhibiting clear disease symptoms, which is considered too late and ineffective. This is because *F. oxysporum* of processing tomatoes typically does not exhibit disease symptoms until the middle to late growth stage, in glasshouse bioassay, and disease symptoms would not appear until four weeks after pathogen inoculation. Hence, rapid disease detection in the early stage is favored for more effective and possible disease management.

The early detection of plant diseases is crucial because it is the key to effective disease control. For most soilborne pathogens, once symptoms have developed in a crop, it is already too late for disease management to occur [4,5,6]. As a result, a more effective system is desired to detect plant diseases in a comparatively early stage for the possible implementation of disease management. To achieve this, several different digital technologies have been developed and tested. An electronic nose (e-nose), also recognized as an artificial intelligent nose [7], is one of the emergent digital devices for applications in agriculture [8], food, and forestry [9]. A typical e-nose comprises an array of sensors [10] that are sensitive to different volatile organic compounds (VOC) such as ethanol, methane, and many others, depending on the aims and targets [11,12,13]. It may also have a data-processing and analysis unit containing a specific aroma database [14,15]. These targeted VOCs can be constructed according to individual needs and requirements. When the sensors are exposed to different VOCs, the portable device will generate different curves reflecting the sensors’ responses toward these odors [16,17]. For the detection of pathogens using the electronic nose in particular, a previous study showed that an electronic nose developed by Borowik et al. was used to measure odor emissions from potato dextrose agar for the identification of *Rhizoctonia solani* and *Fusarium oxysporum* in vitro [11], and a separation of clean and infected cultures was achieved. Additionally, the e-nose was also used for the early detection of *Penicillium digitatum* and *Penicillium italicum* in citrus fruits during storage purposes [18], and a clear determination of healthy and infected fruits was completed, which proved the possibility of further applications of this technique. Machine learning (ML) modeling can identify healthy and infected plants [12,19]. Measurements taken using an e-nose are usually simple and non-invasive for most of the electronic noses that have been developed; measurements are taken after the device reached a baseline by calibration prior to each measurement and then placed on top of the target samples [7,18].

This study aimed to provide a system for the rapid and effective early detection of *Fusarium oxysporum* in processing tomatoes in a controlled environment. Since the previous identification and detection of *F. oxysporum* have relied on molecular technologies, as previously mentioned, applying an e-nose coupled with ML models has never been implemented before [1,2,3]. Output data from an e-nose was used as inputs to construct more accurate supervised ML classification and regression models to classify tomato plants and soil samples into different levels of pathogen infection and to predict plant physiological parameters using a logistic regression model. The practical implementation of this model provides the industry and growers with a precise and cost-effective early detection option which will facilitate the early adoption of management strategies in the future, considering the increased disease incidence and severity caused by soilborne pathogens.

## 2. Materials and Methods

### 2.1. Plant and Pathogen Preparation

*Fusarium oxysporum* strain UMT01, isolated from diseased plants collected from processing tomato fields in early 2022, was used in this study. Tomato seedlings rated susceptible to *F. oxysporum* [1,2] were raised in individual seedling cells in seed raising mix (Plugger 111, Australian Growing Solutions Pty Ltd, Tyabb, VIC, Australia) for three to four weeks until reaching the two-leaf stage. They were gently removed from the seedling cells, and soil particles were thoroughly washed off the root system. Roots were trimmed and dipped into spore suspensions with different concentrations of *F. oxysporum*; the concentrations of spore suspensions included: (i) 10^2^ spores ml ^−1^ (Low), (ii) 10^4^ spores ml ^−1^ (Medium), (iii) 10^6^ spores ml ^−1^ (High), and (iv) 5 × 10^6^ spores ml ^−1^ (Very high). Additionally, a negative control group with wounded seedlings dipping in sterile water (H_2_O) was set up for comparison. Seedlings with different treatments were transplanted into 1.5 L sterile pots containing sterilized potting mix soil (Plugger 111, Australian Growing Solutions Pty Ltd.), consisting of 90% composted substances, coarse bark, and 10% sand. 

The experiment was conducted in the University of Melbourne glasshouse complex with 12 h of daylight at 25 °C and 12 h of darkness at 20 °C for eight weeks. All pots were properly cleaned and sterilized, and plants were watered every other day to maintain a roughly at-field capacity to avoid waterlogging or drought conditions. A complimentary Osmocote fertilizer was applied according to the recommended rate to supply essential nutrients. The experiment had five replicates with five treatments (four different levels of *F. oxysporum* inoculum and a control treatment with no pathogen), and a total number of 25 pots were set. All pots were arranged in a completely randomized design in a large growth chamber. The experiment was repeated twice to confirm the replicability of the results.

### 2.2. Physiological Measurements

Plant physiological parameters, including stomatal conductance (gs; mol H_2_O m^−2^ s^−1^), transpiration (E; mmol H_2_O m^−2^ s^−1^), and photosynthesis (A; μmol CO_2_ m^−2^ s^−1^), were measured using a Li-6400 XT open gas exchange system (Li-Cor Inc., Environmental Sciences, Lincoln, NE, USA). Measurements were taken from the youngest fully expanded leaves and repeated twice in different tillers of each plant (*n* = 10 per treatment, *n* = 50 in total). Physiological measurements were taken every two weeks throughout the glasshouse pathogenicity test, starting from week two due to fragile seedlings with very small leaf sizes in week 0/day 0 (the day of transplanting), and every fortnightly until week 8, i.e., weeks 2, 4, 6, and 8.

### 2.3. Low-Cost Electronic Nose Measurements 

**Figure 1:**
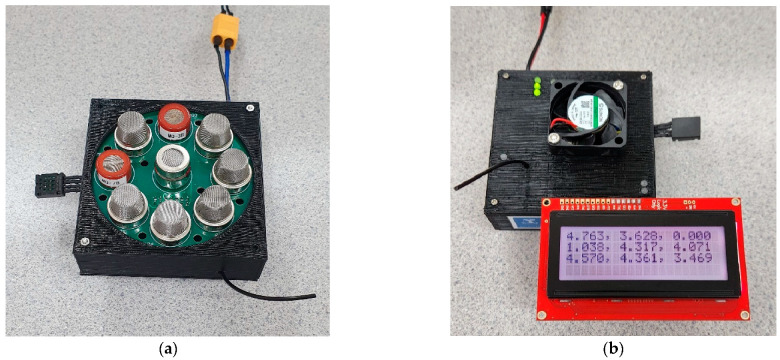
Low-cost electronic nose used in this study showing (**a**) the different sensors and (**b**) extracting fan and liquid-crystal display (LCD) screen for data monitoring. The e-nose has connectivity for an external battery and wireless data transmission capabilities.

A portable and low-cost electronic nose (Figure 1) developed by the Digital Agriculture Food and Wine Group from the University of Melbourne (DAFW-UoM) was used to assess the production of volatile organic compounds [20,21,22,23,24] from processing tomato plants with different treatments and a control. This e-nose consists of an array of nine sensors that are sensitive to different volatile compounds: (i) MQ3 (alcohol), (ii) MQ4 (methane: CH4), (iii) MQ7 (carbon monoxide: CO), (iv) MQ8 (hydrogen: H2), (v) MQ135 (ammonia/alcohol/benzene), (vi) MQ136 (hydrogen sulfide: H_2_S), (vii) MQ137 (ammonia: NH_3_), (viii) MQ138 (benzene/alcohol/ammonia), and (ix) MG811 (carbon dioxide: CO_2_), as well as a humidity and temperature sensor to measure the ambient conditions (Henan Hanwei Electronics Co., Ltd., Henan, China). The e-nose was returned to baseline for 30 s before and after each measurement. The device was placed on top of each plant to record data for 2 min. For each measurement, the e-nose generated outputs every 0.5 s, so a total of 240 readings from each sensor were obtained, totaling 2160 readings from nine sensors for one plant, which was the raw size of the database. Once the e-nose was turned on, and measurements were initiated, the sensor temperature remained constant through all measurements. The e-nose was used to measure plants when the soil was exposed and covered using plastic wrap to find any soil-pathogen-host interactions. The output data (volts) were then extracted and analyzed using a code written in Matlab^®^ R2020a (Mathworks Inc., Natick, MA, USA) by the DAFW-UoM, to extract the mean values of ten segments from the highest peak of the curves, the highest and most stable time of the e-nose curves, as described by Gonzalez Viejo et al. [19]. As 10 mean values were extracted for the nine sensors, a total of 90 readings were extracted, which was the size of the database after extraction (Supplementary Material Figure S1). The e-nose measurements were taken from week 0/day 0, i.e., the day of transplanting, and week 0/day 3, followed by four fortnightly measurements throughout the glasshouse pathogenicity test (weeks 2, 4, 6, and 8), which were co-measured with the plant physiological parameters, as described in 2.2.

### 2.4. Parallel Soil Experiment

A parallel experiment was conducted to determine if the electronic nose was able to detect any interactions or changes in the production of VOCs in the soil after being inoculated with different levels of *F. oxysporum* spores. For this purpose, steam sterilized potting mix was put into 1.5 L pots that were cleaned using the same protocol described in 2.1. The pots were prepared with a sterile potting mix without any plant materials. The same treatments of pathogen inoculum were used, i.e., 10^2^ mL^−1^, 10^4^ mL^−1^, 10^6^ mL^−1^, and 5 × 10^6^ mL^−1^, plus a control group with sterile water only. This parallel experiment was set up under the same condition as the pathogenicity test, with a replicate number of five and a total number of 25 pots. 

A true baseline measurement was taken before adding and mixing the relevant spore suspensions of *F. oxysporum* to the potting mix for all 25 pots using the portable e-nose, marked as week 0/day 0. The same measurements were repeated two days later, marked as week 0/day 2, followed by four-week weekly measurements. 

### 2.5. Statistical Analysis and Machine Learning Models

Plant above-ground height, root dry weight, physiological and e-nose data were analyzed using ANOVA and Tukey honestly significant difference (HSD) *post hoc* tests (α = 0.05) using Minitab 2019 (Minitab, LLC, State College, PA, USA) to assess significant differences between treatments. These data were also analyzed for significant correlations (*p* < 0.05) based on covariance using Matlab^®^ R2020a and represented in a matrix.

**Figure 2:**
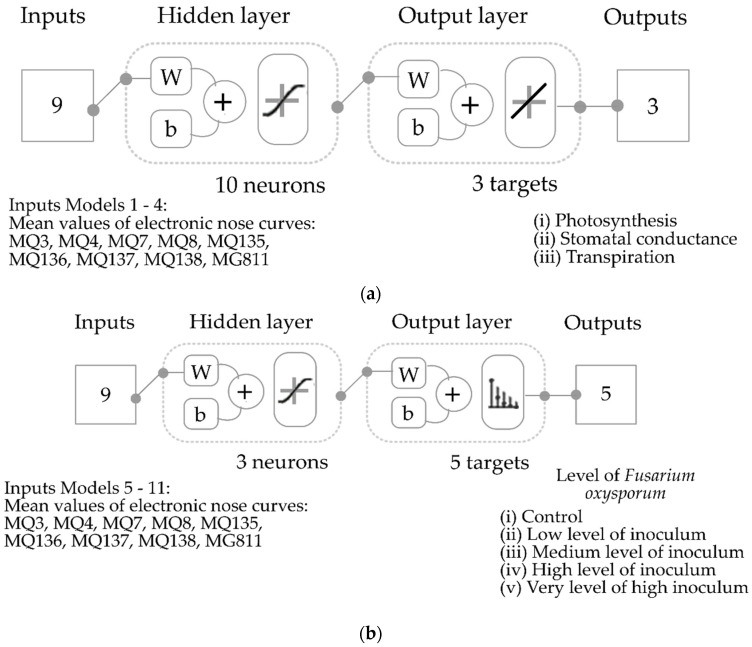
Diagrams of machine learning models based on artificial neural networks showing (**a**) the regression models 1–4, (**b**) pattern recognition models 5–11. Abbreviations: W: weights and b: bias; MQ3: alcohol; MQ4: methane; MQ7: carbon monoxide; MQ8: hydrogen; MQ135: ammonia/alcohol/benzene; MQ136: hydrogen sulfide; MQ137: ammonia; MQ138: benzene/alcohol/ammonia; MG811: carbon dioxide.

Although there are many ML methods, such as support vector machine, random forest, and linear discrimination, which are simpler than ANN, they are, however, usually lower in performance and accuracy and slower in the prediction for deployment [12,19,25,26,27]. Furthermore, methods such as Random Forest and SVM work better with trivial and less intensive data [27]. Furthermore, for regression models, ANN is the only method that supports multiple targets; it predicts multiple variables simultaneously, which is one of the main advantages of regression ANN models over other machine learning methods [28]. Four ML regression models based on artificial neural networks were developed to predict plant physiological data: (i) photosynthesis, (ii) stomatal conductance, and (iii) transpiration, using the ten mean values of ten segments in the e-nose curves as inputs. Different models were constructed using data from all treatments for weeks 2, 4, 6, and 8 (models 1–4; Figure 2a). All models were developed using a customized code written in Matlab^®^ R2020a by the DAFW-UoM to test 17 training algorithms in a loop and to find the best models based on accuracy and performance. The Bayesian regularization training algorithm rendered the most accurate models with no signs of under- or overfitting. Data were divided randomly into 70% for training and 30% for testing using a performance algorithm based on the means squared error [29].

Seven pattern recognition ML models (Figure 2b) were developed to classify tomato plants and soil samples into different levels of *F. oxysporum* infection: (i) control, (ii) low (10^2^ spores mL^−1^), (iii) medium (10^4^ spores mL^−1^), (iv) high (10^6^ spores mL^−1^), and (v) very high (5 × 10^6^ spores mL^−1^), using the ten mean values of ten segments in the e-nose curves as inputs. Different models were developed for different dates at day 0 + day 3, weeks 2, 4, 6, and 8 (models 5–9, respectively) for tomato plants, and day 0 + day 2 (model 10) and weeks 1–4 for soil samples (model 11). The Bayesian regularization training algorithm rendered the most accurate models with no signs of under- or overfitting. Data were divided randomly into 70% for training and 30% for testing using the MSE performance algorithm. 

## 3. Results

Table 1 shows the significant differences (*p* < 0.05) in photosynthesis, stomatal conductance, and transpiration starting from week 2. Photosynthesis for the control at week 6 was significantly higher (17.84 μmol CO_2_ m^−2^ s^−1^) than the inoculated treatments (2.49–10.42 μmol CO_2_ m^−2^ s^−1^). Unlike photosynthesis, stomatal conductance at week 2 and week 8 was significantly higher than the inoculated plants (1.03 and 0.98 mol H_2_O m^−2^ s^−1^, respectively). Lastly, for transpiration, week 4 and week 6 of the control plants had significantly higher values than the inoculated treatments (6.60 and 6.46 H_2_O m^−2^ s^−1^, respectively). All inoculated processing tomatoes did not exhibit visible diseased symptoms until four weeks after inoculation, although the physiological response started to show significant differences from week 2. In general, the control plants had the highest gas exchange rates for all three parameters, and as the pathogen inoculum gradually increased, all three parameters decreased significantly.

**Figure 3:**
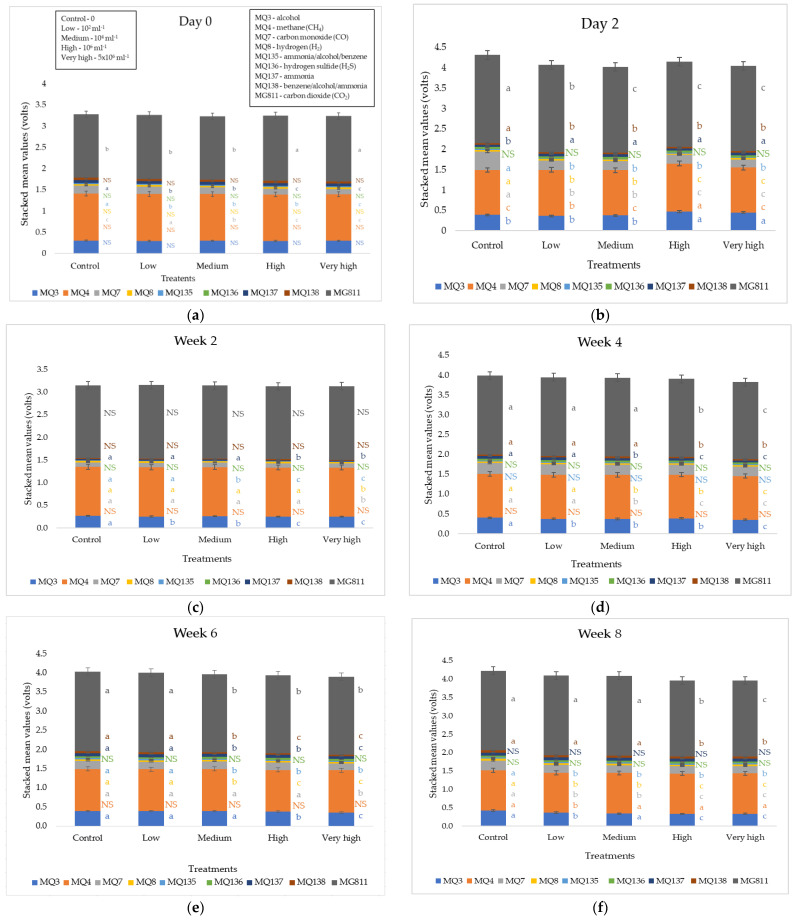
Electronic nose outputs (stacked mean values) for all treatments at each measurement of the glasshouse pathogenicity experiment for (**a**) day 0, (**b**) day 2, (**c**) week 2, (**d**) week 4, (**e**) week 6, and (**f**) week 8. Error bars represent 95% confidence intervals, and columns that do not share the same letters are significantly different (*p* < 0.05).

Figure 3 shows stacked mean values from the e-nose sensor readings for all treatments. There were no significant differences (*p* > 0.05) at day 0 except for MG811 (CO_2_), MQ7 (CO), MQ135 (ammonia/alcohol/benzene), and MQ137 (ammonia), as the measurements were taken right after the transplanting of seedlings; hence no inoculation had occurred yet. On day 3, significant differences (*p* < 0.05) were observed for most of the sensors except for MQ136 (H_2_S). For weeks 2–8, significant differences were found in most sensors except MQ136 (H_2_S) in all measurement weeks, MG811 (CO_2_) at week 2, MQ138 (benzene/alcohol/ammonia) at week 2, MQ4 (CH_4_) at weeks 2, 4 and 6, MQ135 (ammonia/alcohol/benzene) at week 4, and MQ137 (ammonia) at week 8. Among the nine sensors, MQ4 (CH_4_) and MQ3 (alcohol) produced the highest mean values. Sensor MG811 (CO_2_) provides inverse outputs; therefore, higher Volts mean lower CO_2_. Overall, the control was the highest in all sensors and significantly different from day 3.

**Figure 4:**
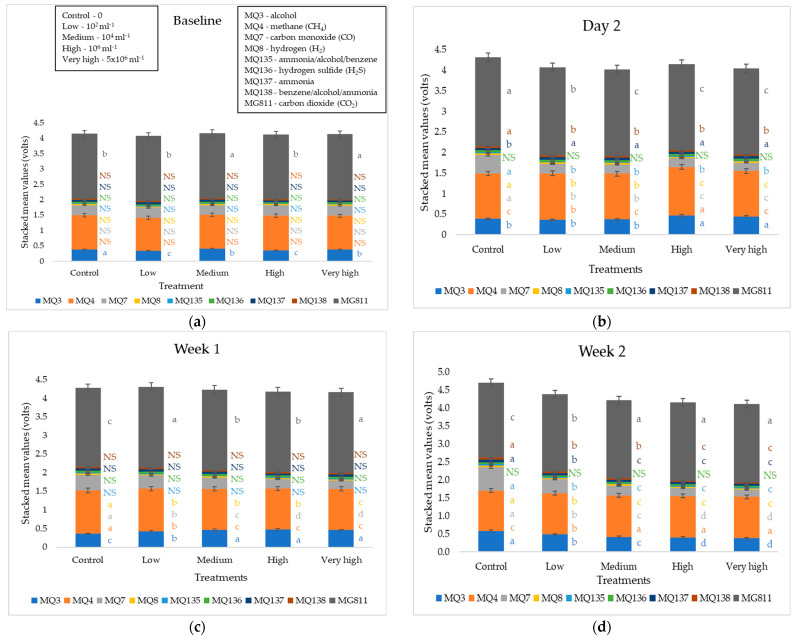

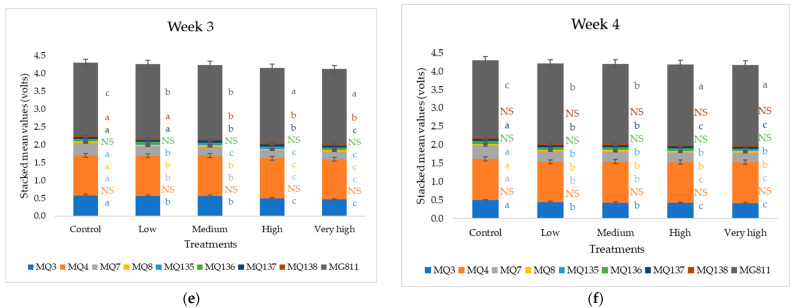
Electronic nose outputs (stacked mean values) for all treatments at each measurement of the parallel soil experiment (**a**) baseline, (**b**) day 2, (**c**) week 1, (**d**) week 2, (**e**) week 3, and (**f**) week 4. Error bars represent 95% confidence intervals, and columns that do not share the same letters are significantly different (*p* < 0.05).

Figure 4 shows the stacked mean values of the e-nose sensor readings for the parallel soil experiment. There were no significant differences (*p* > 0.05) in baseline measurements except for MG811 (CO_2_) and MQ3 (alcohol). Starting from day 2, significant differences were observed for most sensors except MQ136 (H_2_S) in all measurements, MQ138 (benzene/alcohol/ammonia) at weeks 1 and 4, MQ137 (ammonia) at week 1, MQ135 (ammonia/alcohol/benzene) at week 1, and MQ4 (CH_4_) at weeks 3 and 4. Similar to the plant measurements, MQ4 (CH_4_) and MQ3 (alcohol) produced the highest mean values for the soil samples. Control samples had the highest values for MQ7 and MQ8 since day 2 and MQ137 from week 2. On the other hand, treatments 10^6^ and 5 × 10^6^ were the highest in MQ3 on day 2 and week 1 and MQ4 on week 2.

**Figure 5:**
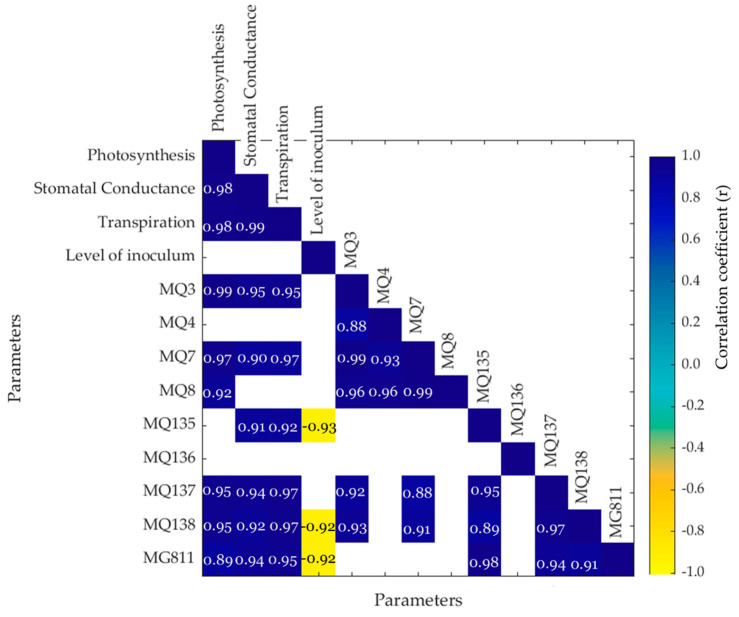
Matrix showing the significant correlations (*p* < 0.05) between the physiological data, level of pathogen inoculum, and the electronic nose sensors. The color bar represents the negative (yellow) to positive (blue) correlations, and the numbers within each box are the correlation coefficients (r).

Figure 5 shows that both photosynthesis and transpiration had a significantly positive correlation (*p* < 0.05) with MQ3 (alcohol; r = 0.99 and r = 0.95, respectively), MQ7 (CO; r = 0.97 and r = 0.91, respectively), MQ137 (ammonia; r = 0.95 and r = 0.97, respectively), MQ138 (benzene/alcohol/ammonia; r = 0.95 and r = 0.97, respectively), and MG811 (CO_2_; r = 0.89 and r = 0.95, respectively). Furthermore, MQ135 (ammonia/alcohol/benzene), MQ138 (benzene/alcohol/ammonia), and MG811 (CO_2_) had a significantly negative correlation with the level of inoculum (r = −0.93, r = −0.92, and r = −0.92, respectively). Similarly, stomatal conductance had a positive correlation with MQ3 (r = 0.95), MQ7 (r = 0.90), MQ135 (r = 0.91), MQ137 (r = 0.94), MQ138 (r = 0.92), and MG811 (r = 0.94). Additionally, transpiration and stomatal conductance also had a significantly positive correlation with MQ3 (r = 0.95 for both), MQ7 (r = 0.90 and r = 0.91, respectively), MQ135 (r = 0.91 and r = 0.92, respectively), MQ137 (r = 0.94 and r = 0.97, respectively), MQ138 (r = 0.92 and r = 0.97, respectively), and MG811 (r = 0.94 and r = 0.95 respectively). On the contrary, the level of inoculum had a negative correlation with MQ135 (ammonia/alcohol/benzene, r = −0.93), MQ138 (benzene/alcohol/ammonia, r = −0.92), and MG811 (CO_2_, r = −0.92).

Table 2 shows the results from the machine learning regression models to predict plant physiological data (photosynthesis, stomatal conductance, and transpiration) using the e-nose outputs as inputs. Models 1–4 were constructed using data from all treatments from weeks 2–8. All models had very high accuracies and slope (b) values with R = 0.97 and b = 0.99 for model 1, R = 0.98 and b = 0.97 for model 2, R = 0.99 and b = 0.98 for model 3, and R = 0.97 and b = 1.00 model 4. For all four models, no signs of under- or overfitting were observed since MSE for training (<0.01 for all) was lower than the testing (≤0.02 for all). 

**Figure 6:**
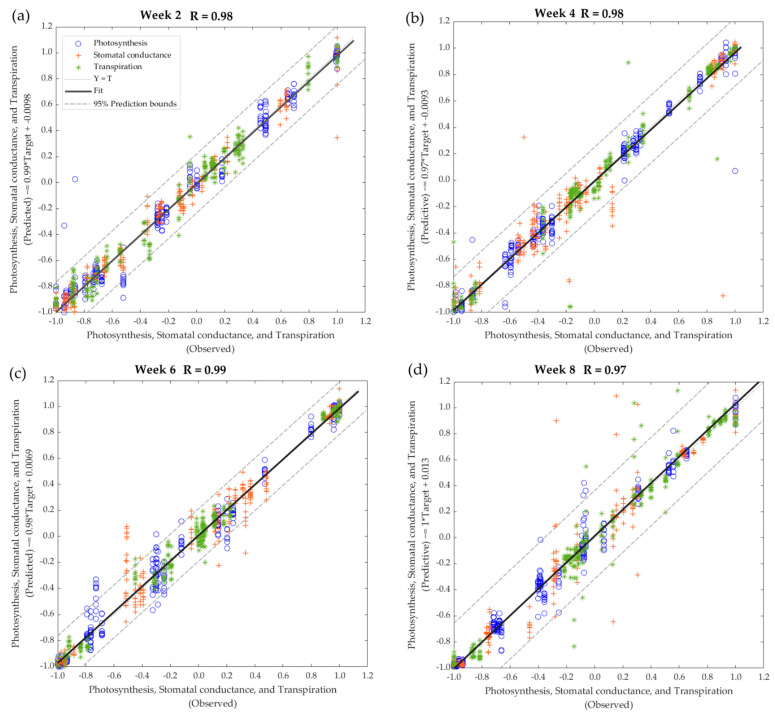
Overall regression models for the glasshouse pathogenicity bioassay of the processing tomato plants to predict plant physiological data using the electronic nose outputs as inputs for general data using all treatments at (**a**) week 2, (**b**) week 4, (**c**) week 6, and (**d**) week 8. Abbreviations: R: correlation coefficient; T: targets.

Figure 6 shows that, according to the 95% prediction bounds, model 1 had 2.13% as outliers (16 out of 250 data points), with the majority from photosynthesis (50% out of 16 outliers). Model 2 presented 2.53% as outliers (19 out of 750 data points), with most from stomatal conductance (57.89% out of 19 outliers). On the other hand, model 3 had 4.93% as outliers (37 out of 750 data points), with stomatal conductance and photosynthesis presenting the highest number of outliers (each with 45.95% out of 37 outliers). Furthermore, model 4 had 3.20% as outliers (24 out of 750 data points), with most from transpiration (41.67% out of 24 outliers).

Table 3 shows the results from the pattern recognition models to classify tomato plants into five different inoculum levels. Models 6 (week 2) and 8 (week 6) had the highest overall accuracy of 96.8% for both, followed by model 5 (day + day 3) with 95.2% and models 9 (week 8) and 7 (week 4) with 94.8% and 94.4%, respectively. None of the five models showed signs of under- or overfitting since the MSE values for training (<0.01 for all) were lower than the testing stage (≤0.02).

Table 4 shows the results from the pattern recognition models to classify soil samples into five different treatments. It can be observed that both models 10 and 11 had high overall accuracy. Model 10 had a slightly higher overall accuracy (96.22%) than model 11 (94.81%). Neither of the models had any signs of under- or overfitting since the MSE values for training (<0.01 for both) were lower than the testing (0.02 for both).

**Figure 7:**
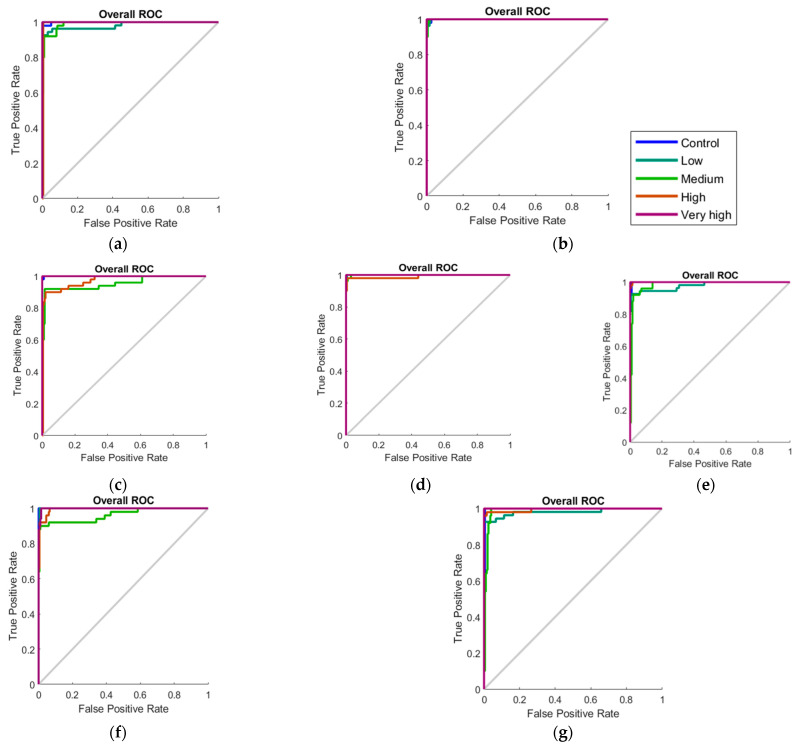
Overall receiver operating characteristic plot for the pattern recognition models of the glasshouse pathogenicity bioassay (models 5–9, **a**–**e**, respectively) and the parallel soil experiment (models 10 and 11, **f** and **g**, respectively).

Figure 7 shows the overall receiver operating characteristic [30] curves of the pattern recognition models for the classification of the tomato plants [31] and soil samples (f and g), which assessed the overall diagnostic performance of the pattern recognition models. As true-positive was achieved for all pattern recognition models, the most optimal models, as described before (models 5–11), were selected.

## 4. Discussion

### 4.1. Physiological Response of Tomato Plants to F. oxysporum Infection

The reduction in plant physiological parameters may have resulted from water and nutrient deficits due to roots being infected by *Fusarium oxysporum* [32,33,34,35]. Since the Australian Processing Tomato Industry has a normal growing season of four months, and physiological data started to differ significantly from week 2, the early change in plant physiological response was important for the further application of early detection. Transpiration and photosynthesis were found to be positively correlated with stomatal conductance and stomata opening. The results were consistent with previous research where root systems were infected by the collar and root rot *Fusarium oxysporum*, leading to a damaged collar and root system with lesions and rot [1,32,35]; hence effective water and nutrients uptake from the root system was significantly reduced, and stomatal closure occurred as a stress response resulting from water deficit [36,37,38]. Anatomical alterations to smaller stomatal sizes and stomatal closure due to water deficit caused by poor root growth leads to decreased stomatal conductance [39]. Gudesblat et al. [40] and Ye et al. [41] showed that once stomatal activities were limited in response to increased pathogen inoculum, especially for pathogenic fungal species, the photosynthetic capacity, transpiration, and gas exchange rates were all significantly reduced. 

### 4.2. Production of Plant Volatile Compounds in Response to F. oxysporum Infection 

For the e-nose readings, the variability in the production of VOCs was expected, which was assumed to be correlated with the interactions between *F. oxysporum* and the tomato plants. As mentioned previously, the normal growing season of Australian processing tomatoes is four months, and significant differences in e-nose results were observed from day 3 of processing tomatoes; hence the early detection of changes in VOC production was achieved. For the volatile compound MQ3 (alcohol), previous research found that *F. oxysporum* was able to assimilate ethanol from plant cell walls [42,43,44,45,46]. However, this experiment found that with increased *F. oxysporum* inoculum, the production of alcohol decreased gradually. The largely reduced photosynthesis could have caused this variability hence the reduced biomass production and depletion of available resources and nutrients, leading to a significantly reduced alcohol production with an increased pathogen inoculum. 

The concentration of MG811 (CO_2_) was consistent with the physiological data with high levels of *F. oxysporum* inoculum causing root infection leading to significant stomatal closure due to water deficit, thereby largely limiting gas exchange. This is similar to previous studies [32,33], where the *Fusarium* crown and root rot of tomatoes caused stomatal closure due to water deficit as a result of root lesions and severely damaged water-conducting tissues. Consequently, plants were unable to capture and utilize CO_2_ from the ambient environment to achieve effective gas exchange with low stomatal conductance and photosynthesis [47,48], which could lead to the accumulation and elevated levels of CO_2_ [49,50,51] on the plant leaf surface being captured by the e-nose.

### 4.3. Response of Soil Samples to F. oxysporum Inoculation

In general, the e-nose readings from soil samples were not as responsive as from the tomato plants, and the differentiation or identification of soil samples with different concentrations of inoculum was unsuccessful. For the e-nose outputs of soil samples, the fluctuation at the baseline measurement for MQ3 (alcohol) and MG811 (CO_2_) could have been due to the high sensitivity of the e-nose that took measurements every 0.5 s, hence able to capture any changes in volatile compounds from the soil surface [19]. As the experiment progressed, the higher release of CO_2_ with increased inoculum levels could have been due to the ability of *F. oxysporum* to interact with the organic compounds in the potting mix, hence assimilating and releasing CO_2_ back to the environment, which was consistent with previous studies [51,52,53,54]. For MQ3 (alcohol), previous studies found that *F. oxysporum* was able to ferment ethanol directly from monosaccharides in plant cell wall tissues [42,43,44,45,46] under certain anaerobic and microaerobic conditions [44,45]. Nonetheless, as *F. oxysporum* gradually became dominant in the microbial environment, the resources and nutrients were largely depleted; thus, ethanol fermentation may not have continued further, and a reduction in alcohol production was observed after week 2. As a result, the e-nose could be used as an indicator to identify clean, healthy soil from *F. oxysporum*-infected soil; however, the identification of different inoculum levels cannot be completely achieved solely based on e-nose results as there was no statistical difference in the inoculated treatments with different levels of infection. Determination of the exact concentration of the inoculum in the soil over time (using qPCR to detect the pathogen’s DNA) would provide a more accurate method to correlate volatile compounds with the level of inoculum in the soil. 

### 4.4. Development of Machine Learning Models

The glasshouse pathogenicity bioassays were conducted in a growth chamber with highly controlled ambient conditions; hence the models developed did not include disturbance from any other abiotic or biotic stressors. The high similarity of ML models developed from the glasshouse pathogenicity bioassay and the parallel soil experiment demonstrated the accuracy of the novel low-cost digital e-nose compared to more sophisticated and expensive instrumentations, such as gas chromatography [55,56,57] and/or near-infrared spectroscopy [58]. 

Compared to the growing season of Australian processing tomatoes, normally three to four months, the first 1.5–2 months after sowing or transplanting (depending on different planting methods) are extremely crucial when considering disease management. The eight-week glasshouse pathogenicity bioassay provided an effective way for early disease detection, as both pattern recognition and regression models had very high accuracies from week 2. For the glasshouse pathogenicity bioassay, the e-nose outputs, machine learning regression, and pattern recognition models provided significant separation and prediction of plant physiological parameters and levels of infection, respectively, with high accuracies. Considering the parallel soil experiment, the separation of soil samples inoculated with different levels of *F. oxysporum* was not completely successful when based on e-nose readings alone, and simple statistics such as ANOVA and the ANN pattern recognition models for classification purposes had a very high accuracy of 96.22% and 94.81%, respectively (models 10 and 11). As mentioned previously, all models developed from tomato plants based on e-nose outputs could demonstrate the differences in the pathogen–plant interactions under different inoculum levels, which were successfully validated from the Licor data. The plant physiological machine learning models developed from the e-nose outputs as inputs and Licor data as targets for all treatments had high correlation coefficients and no sign of overfitting. 

For both ML regression and pattern recognition models, model 1 (regression model, week 2) with a correlation coefficient R = 0.97, models 2 and 5 (pattern recognition models, day 0 + day 3 and baseline + day 2, respectively), with an overall accuracy of 95.2% and 96.22%, respectively, were extremely crucial as they could be considered as a valid early detection of *F. oxysporum* infection on both tomato plants and soil samples. Since no visible or clear disease symptoms had developed in such a short period, timely effective management strategies could be implemented once early detection is achieved through different machine learning models. As the models were developed from a highly controlled environment that focused only on the impact of the soilborne pathogen *F. oxysporum*, the next stage would be to implement this technique and to deploy the models in field conditions where there will be more complicated ambient conditions with more complex soil–microbial–host interactions. The low-cost, effective, and accurate e-nose coupled with ML modeling could be further applied to monitor pathogen interactions with plants and/or soil through integrated disease management. 

## 5. Conclusions

This is the first report of applying the low-cost electronic nose and machine learning modeling for the early detection of disease caused by *Fusarium oxysporum* in processing tomato plants. With the accurate machine learning models developed from the cost-effective portable electronic nose, it provides solid evidence and validation for the application of the e-nose in processing tomato fields for the early detection of plant disease and the assessment of plant health status without destructive sampling. The early detection of changes in the production of volatile compounds could hence be applied in field conditions to identify early signs of plant diseases and pathogen infection. The decrease in gas exchange could provide preliminary evidence of compromised plant health conditions, especially in the early growth stages. In addition, the ML models with high accuracy can also be further applied to assess and evaluate other abiotic and biotic stressors related to poor plant growth, including, but not limited to, water stress, droughts, insects, or other soilborne and foliar pathogens. This provides an efficient and cost-effective plant monitoring system that could become one of the important aspects of integrated pest and disease management.

## Figures and Tables

**Table 1:** Mean values ± standard error of the plant physiological data for all treatments.

Treatment	Photosynthesis (μmol CO_2_ m^−2^ s^−1^)				Stomatal Conductance (mol H_2_O m^−2^ s^−1^)				Transpiration (mmol H_2_O m^−2^ s^−1^)			
Measurements	W2	W4	W6	W8	W2	W4	W6	W8	W2	W4	W6	W8
Control	14.86 ^a^	14.17 ^a^	17.84 ^a^	15.42 ^a^	1.03 ^a^	0.94 ^a^	0.93 ^a^	0.98 ^a^	5.19 ^ab^	6.60 ^a^	6.46 ^a^	5.61 ^a^
	±0.57	±0.11	±0.43	±0.07	±0.12	±0.01	±0.01	±0.04	±0.42	±0.13	±0.05	±0.08
10^2^ (low)	10.98 ^b^	10.42 ^b^	11.77 ^b^	9.41 ^b^	0.77 ^ab^	0.44 ^bc^	0.68 ^b^	0.57 ^b^	4.68 ^b^	4.55 ^b^	4.20 ^b^	4.47 ^b^
	±0.84	±0.16	±0.54	±0.14	±0.05	±0.23	±0.02	±0.27	±0.15	±0.36	±0.09	±0.14
10^4^ (medium)	6.32 ^c^	9.75 ^b^	8.28 ^bc^	6.60 ^c^	0.37 ^bc^	0.38 ^bc^	0.59 ^b^	0.47 ^bc^	3.31 ^c^	4.11 ^b^	3.89 ^bc^	3.41 ^c^
	±0.48	±0.13	±0.11	±0.08	±0.05	±0.23	±0.02	±0.04	±0.20	±0.12	±0.03	±0.11
10^6^ (high)	4.89 ^cd^	6.03 ^d^	4.43 ^cd^	3.55 ^dc^	0.36 ^bc^	0.31 ^bc^	0.34 ^bc^	0.23 ^c^	2.92 ^cd^	3.45 ^c^	3.07 ^c^	3.05 ^c^
	±0.42	±0.23	±0.16	±0.14	±0.03	±0.10	±0.01	±0.03	±0.18	±0.15	±0.13	±0.04
5 × 10^6^ (very high)	2.29 ^d^	2.26 ^d^	2.49 ^d^	1.07 ^d^	0.20 ^c^	0.16 ^c^	0.13 ^c^	0.11 ^c^	2.56 ^d^	2.40 ^d^	1.39 ^d^	1.27 ^d^
	±0.41	±0.16	±0.10	±0.07	±0.02	±0.01	±0.02	±0.01	±0.20	±0.09	±0.11	±0.09

Different letters a–d show significant differences between treatments (rows) based on ANOVA and the Tukey honestly significant difference (HSD) post hoc tests (α = 0.05; *p* < 0.05).

**Table 2:** Machine learning regression models based on artificial neural networks (Bayesian regularization) to predict plant physiological data using the electronic nose outputs as inputs; ten neurons were used. Abbreviations: R: correlation coefficient; b: slope; MSE: means squared error.

Stage	Samples	Observations	R	b	Performance (MSE)
Model 1—All treatments, Week 2					
Training	175	525	0.99	0.98	<0.01
Testing	75	225	0.95	0.99	0.02
Overall	250	750	0.97	0.99	-
Model 2—All treatments, Week 4					
Training	175	525	0.99	0.99	<0.01
Testing	75	225	0.93	0.93	0.02
Overall	250	750	0.98	0.97	-
Model 3—All treatments, Week 6					
Training	175	525	0.99	0.98	<0.01
Testing	75	225	0.98	0.97	0.01
Overall	250	750	0.99	0.98	-
Model 4—All treatments, Week 8					
Training	175	525	0.99	0.99	<0.01
Testing	75	225	0.91	1.00	0.02
Overall	250	750	0.97	1.00	-

**Table 3:** Machine learning pattern recognition models based on artificial neural networks (Bayesian regularization) to classify tomato plants into different levels of pathogen inoculum using the electronic nose outputs as inputs. Abbreviations: MSE: means squared error.

Stage	Samples	Accuracy	Error	Performance (MSE)
Model 5—Day 0 + Day 3				
Training	350	99.40%	0.60%	<0.01
Testing	150	85.30%	14.70%	0.02
Overall	500	95.20%	4.80%	-
Model 6—Week 2				
Training	175	98.30%	1.70%	<0.01
Testing	75	93.30%	6.70%	0.02
Overall	250	96.80%	3.20%	-
Model 7—Week 4				
Training	175	98.30%	1.70%	<0.01
Testing	75	85.30%	14.70%	0.01
Overall	250	94.40%	5.60%	-
Model 8—Week 6				
Training	175	97.70%	2.30%	<0.01
Testing	75	94.70%	5.30%	0.01
Overall	250	96.80%	3.20%	-
Model 9—Week 8				
Training	175	99.40%	0.60%	0.01
Testing	75	84.00%	16.00%	0.02
Overall	250	94.80%	5.20%	-

**Table 4:** Machine learning pattern recognition models based on artificial neural networks (Bayesian regularization) to classify soil samples into different levels of pathogen infection using the electronic nose outputs as inputs. Abbreviations: MSE: means squared error.

Stage	Samples	Accuracy	Error	Performance (MSE)
Model 10—Baseline + Day 2				
Training	350	98.30%	1.70%	<0.01
Testing	150	91.35%	8.65%	0.02
Overall	500	96.22%	3.78%	-
Model 11—Weeks 1–4				
Training	700	97.13%	2.87%	<0.01
Testing	300	89.40%	10.60%	0.02
Overall	1000	94.81%	5.19%	-

## Data Availability

Data and intellectual property belong to the University of Melbourne; any sharing needs to be evaluated and approved by the university.

## Supplementary Materials

The following supporting information can be downloaded at: https://www.mdpi.com/article/10.3390/s22228645/s1, Figure S1: Simplified flowchart showing e-nose data acquisition and processing for each individual plant.
