# Supplementary material for: Early Detection of Fusarium oxysporum Infection in Processing Tomatoes (Solanum lycopersicum) and Pathogen–Soil Interactions Using a Low-Cost Portable Electronic Nose and Machine Learning Modeling
Source: Sensors (Basel). 2022 Nov 9;22(22):8645. doi: 10.3390/s22228645 (PMC9693623; doi:10.3390/s22228645)
Supplement: Supplementary file 1 [file sensors-22-08645-s001.zip › sensors-1997183-supplementary.pdf]

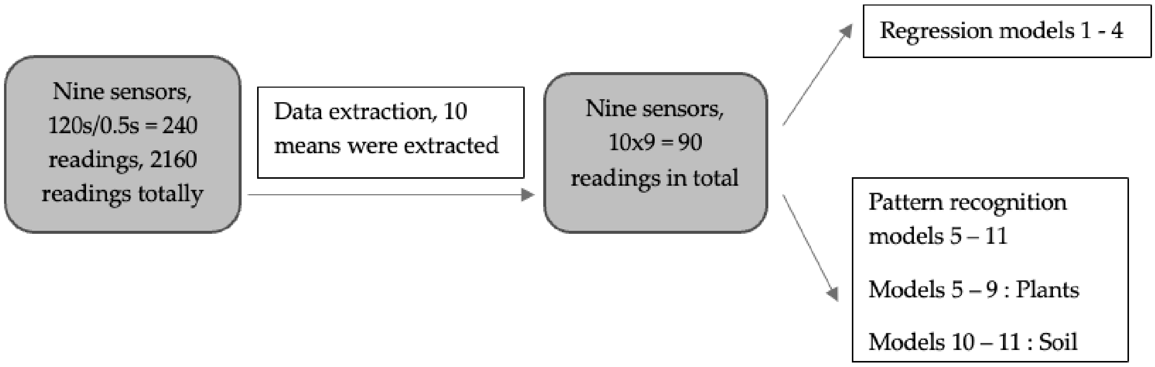

Figure S1. Simplified flowchart showing e-nose data acquisition and processing for each individual plant.
